# Feasibility and usefulness of cognitive monitoring using a new home-based cognitive test in mild cognitive impairment: a prospective single arm study

**DOI:** 10.1186/s12877-024-04850-4

**Published:** 2024-03-08

**Authors:** Kyung Won Lee, Yun Jeong Hong, Eun Jin Yang, Si Baek Lee, Seong Hoon Kim, Seunghee Na, Young-Do Kim, Jeong Wook Park

**Affiliations:** 1 Department of Neurology, Uijeongbu St. Mary’s Hospital, The Catholic University of Korea, 11765 Seoul, Korea; 2 Department of Neurology, Incheon St. Mary’s Hospital, The Catholic University of Korea, Seoul, Korea

**Keywords:** Cognitive monitoring, Validation, Telephone-based cognitive test, Mild cognitive impairment, Alzheimer’s disease

## Abstract

**Background:**

The risk of dementia is increased in subjects with mild cognitive impairment (MCI). Despite the plethora of in-person cognitive tests, those that can be administered over the phone are lacking. We hypothesized that a home-based cognitive test (HCT) using phone calls would be feasible and useful in non-demented elderly. We aimed to assess feasibility and validity of a new HCT as an optional cognitive monitoring tool without visiting hospitals.

**Methods:**

Our study was conducted in a prospective design during 24 weeks. We developed a new HCT consisting of 20 questions (score range 0–30). Participants with MCI (*n* = 38) were consecutively enrolled and underwent regular HCTs during 24 weeks. Associations between HCT scores and in-person cognitive scores and Alzheimer’s disease (AD) biomarkers were evaluated. In addition, HCT scores in MCI participants were cross-sectionally compared with age-matched cognitively normal (*n* = 30) and mild AD dementia (*n* = 17) participants for discriminative ability of the HCT.

**Results:**

HCT had good intra-class reliability (test-retest Cronbach’s alpha 0.839). HCT scores were correlated with the Mini-Mental State Examination (MMSE), verbal memory delayed recall, and Stroop test scores but not associated with AD biomarkers. HCT scores significantly differed among cognitively normal, MCI, and mild dementia participants, indicating its discriminative ability. Finally, 32 MCI participants completed follow-up evaluations, and 8 progressed to dementia. Baseline HCT scores in dementia progressors were lower than those in non-progressors (*p* = 0.001).

**Conclusion:**

The feasibility and usefulness of the HCT were demonstrated in elderly subjects with MCI. HCT could be an alternative option to monitor cognitive decline in early stages without dementia.

**Supplementary Information:**

The online version contains supplementary material available at 10.1186/s12877-024-04850-4.

## Introduction

Mild cognitive impairment (MCI) indicates existence of objective cognitive impairments without limitation in activities of daily living. The risk of dementia is increased in MCI subjects, with clinical progression rates ranging from 10 to 15% annually compared with the annual incidence rate of dementia from 1 to 2% in elderly subjects in the general population. Formal neuropsychological tests in this stage are usually performed using in-person methods with paper and pencil. Despite numerous hospital-based neuropsychological tests, telephone-based cognitive assessments that can be performed at home are lacking [1–3]. Telephone-based cognitive tools were validated in only a few previous studies, which were limited due to small sample size and heterogeneity in the target population, test methods, and different cut-off scores [1–3]. Furthermore, demographic and/or cultural factors were not considered in previous tests, and most were validated in White, English-speaking, and highly educated participants [1]. Remotely administered cognitive monitoring tools validated in Korean elderly subjects that can assess subtle early cognitive decline are necessary.

We hypothesized that a brief home-based cognitive test (HCT) for Korean elderly subjects is needed for regular monitoring of cognitive function without visiting the hospital. In the present study, an HCT was developed, and the correlations with existing hospital-based in-person neuropsychological tests were evaluated for validation.

## Materials and methods

### Subjects

This study was prospectively performed at a university-affiliated dementia clinic from July 2019 to December 2021. Elderly subjects who visited the hospital due to persistent cognitive worsening and who were diagnosed with MCI were consecutively recruited during the study period. Clinical diagnosis of MCI was based on evaluations including detailed neuropsychological test battery named Seoul Neuropsychological Screening Battery (SNSB) [4], brain MRI, and routine blood chemistry including syphilis, thyroid function, vitamin B12/folate/thiamine levels, and apolipoprotein epsilon (APOE) genotyping. SNSB includes tests evaluating attention, language, praxis, visuospatial function, verbal and visual memory, and executive function as well as Korean version of instrumental activities of daily living (K-IADL) [4]. Plasma oligomeric amyloid beta level [5, 6] was examined at baseline. At baseline, MRI results within 1 year and blood test results within 3 months were accepted as the baseline findings. If there were no results within the period, retesting was performed. The study inclusion criteria were the following: (1) any cognitive complaint reported by participant or family members; (2) neuropsychological test shows impairment in at least 1 domain of cognitive function below − 1.0 standard deviation (SD) compared with age-, gender-, and education-adjusted norms; [4] and (3) ADL is normal based on MCI criteria according to Petersen and colleagues [7]. The physicians determined whether ADLs are normal based on K-IADL and Clinical Dementia Rating (CDR) scores [8]. K-IADL score below the cut-off score [9] of 0.43 and CDR score of 0.5 (sum of boxes scores below 3.5) were considered to be preserved ADLs. Exclusion criteria were (1) dementia status; (2) acute poor medical condition that may be associated with cognitive impairments such as acute kidney injury, hepatic encephalopathy, or systemic infection; and (3) participant or a family member refused to participate in the study.

For cross-sectional comparisons of HCT scores between cognitively normal, MCI, and mild Alzheimer’s disease (AD) dementia subjects [10], individuals with mild dementia CDR score 0.5 or 1.0^7^ who fulfilled the diagnostic criteria of National Institute on Aging-Alzheimer’s Association (NIA-AA) workgroups on diagnostic guidelines for AD [10] (*n* = 17) and participants with normal cognition based on SNSB [4] results ( > − 1.0 SD compared with age-, gender-, and education-adjusted norms in all domains; *n* = 30) were additionally enrolled. For cross-sectional assessment of test-retest reliability, 10 of the normal cognition subjects underwent HCT repeatedly with a 1-week interval. For cross-sectional comparison of inter-rater reliability, 2 independent raters examined the HCT in the same participant with normal cognition (*n* = 10) with a 1-week interval.

This study was approved by the institutional review board of Uijeongbu St. Mary’s Hospital (approval number: UC19OESI0069), and written informed consent was obtained from all participants. The study was conducted in accordance with the Declaration of Helsinki and principles of Good Clinical Practice. The personal information of the research participants was managed in an anonymized manner and stored in a location with restricted access except for the researcher in charge.

### HCT monitoring

HCT was developed based on the Korean version of the Mini-Mental State Examination (K-MMSE) [11] to assess general cognitive functions over phone calls. The two tests are similar because they can be performed in approximately 5–10 min and scores range from 0 to 30, with a higher score indicating better cognitive function. However, several differences exist between HCT and K-MMSE; we minimized tests for orientation, increased tests for registration and recall, and added tests for frontal functions because the HCTs should be performed in non-demented participants using phone calls. The HCT was composed of the following: orientation to time (3 questions) and place (3 questions); verbal memory registration (5 words) and free recall (5 words); attentional tests that included serial 7 subtraction and backward digit span (4 numbers, e.g., 3-2-7-3); 2 frontal function tests using Controlled Oral Word Association Test (COWAT) phonemic (2 points) and 1 question for abstract thinking (2 points, e.g., what should you do if your room catches fire? or why do you wash your clothes?); and language function tests including naming (2 questions), comprehension (2 questions), repetition (1 question), and fluency (1 question).To prevent a potential learning effect, 7 sets were prepared for verbal memory registration and recall, language function tests, and tests for abstract thinking, and 1 among them was chosen for each test. Test-retest consistency and inter-rater reliability in the 10 cognitively normal controls were assessed.

### Brain MRI scans

Brain MRI was performed using a 3.0-Tesla scanner (GE Medical Systems, Milwaukee, WI, USA), including fluid attenuated inversion recovery (FLAIR), gradient-echo, and three-dimensional (3D) T1-weighted images. The white matter hyperintensities (WMHs) were rated using a visual rating scale of axial FLAIR images. In brief, periventricular WMHs and deep WMHs were evaluated separately and rated as minimal (grade 1), moderate (grade 2), or severe (grade 3) [12]. Lacunes were defined as small lesions (3–15 mm in diameter) hyperintense on T2- and hypointense on T1-weighted images with a perilesional halo on FLAIR [13]. Cerebral cortical microbleeds were defined as round and homogeneously low-signal lesions < 10 mm in diameter in lobar areas on gradient echo images [13]. Hippocampal atrophy was rated on coronal T1-weighted images using Scheltens’ visual rating scale [14]. The mean of the left and right hippocampal atrophy scores was used. The number of lacunes, number of microbleeds, degree of WMH, and degree of hippocampal atrophy were measured by a neurologist blinded to the data.

### Neuroimaging analysis

Processing and analysis of MRIs were performed using an artificial intelligence (AI)-based program named AQUA 2.0 (Neurophet, South Korea). The details of the MRI analysis including segmentation and normative data analysis were described in previous studies [15]. A normative percentile of the dataset was obtained using the East-Asian database described in a previous report [16] and calculated as the adjusted volume corrected to total intracranial volume as suggested in a previous study [17]. Brain MRI analyses showed regional standardized volumes (percentile) adjusted based on age, gender, and total intracranial volume [18]. 

### Neuropsychological tests

All participants were diagnosed with MCI using the formal neuropsychological test battery SNSB [4], including the K-MMSE, CDR, K-IADL, tests for attention (digit span test), language (Boston Naming Test, tests for comprehension/repetition/fluency), visuospatial function (Rey Complex Figure Test, RCFT), verbal and visual memory function (Seoul Verbal Learning Test, SVLT and RCFT recall test), and frontal executive function (contrasting program, go-no-go, COWAT test, and Stroop test) [4]. Age-, sex-, and education-specific norms were used to interpret the SNSB results. The percentile scores, standardized scores adjusted by age, sex, and education, are based on a large nationwide Korean sample (1100 people), making it possible to perform comparisons with the population averages. Scores ≥ 16th percentile, which were compared to − 1 standard deviation (SD) of the norm, were defined as normal. Scores ≥ 16th percentile, which were comparable to − 1 SD of the norm, were defined as normal. Follow-up neuropsychological evaluations of clinical progression/cognitive decline included SNSB, K-MMSE, CDR, K-IADL, neurological and physical examinations, and history taking to assess clinical progression to dementia. A trained neuropsychologist administered the cognitive tests. Progression to dementia was evaluated based on follow-up neuropsychological tests and history taking in outpatient clinics at endpoint. Participants with CDR sum of boxes score ≥ 3.5 or K-IADL scores ≥ 0.43 were considered to have progressed to dementia.

### Plasma amyloid beta values

Plasma oligomeric amyloid beta values were measured using the Multimer Detection System-oligomeric Aß (MDS-OAß) method [5] at baseline. In brief, the inBloodTM™ OAß test (People Bio Inc., Gyeonggi-do, Republic of Korea) was used to quantify MDS-OAß values in heparin vacutainer tubes; higher values indicate stronger amyloid oligomeric tendencies with vigorous amyloidosis [5, 6]. The plasma amyloid values using MDS-OAß method were higher in AD patients than in normal controls or non-AD dementia patients showing good discriminations [5, 6]. Plasma amyloid beta values correlated well with conventional AD biomarkers including [11]C-Pittsburgh compound B (PIB) positron emission tomography (PET), cerebrospinal fluid Aß42 and phosphorylated tau levels.

### Statistical analysis

Test-retest consistency and inter-rater reliability were assessed using Cronbach’s alpha values. Associations between HCT scores and neuropsychological tests scores were investigated. In addition, whether the HCT scores correlated with AD biomarkers was assessed. To assess relevance of the HCT scores and neuropsychological tests scores/AD biomarkers, partial correlation coefficient adjusted for age, sex, and education was calculated. ANOVA was used to compare continuous variables (demographics, HCT test scores, and biomarker findings) among normal cognition, MCI, and mild dementia patients because basic demographics such as age, gender, and education levels were similar among the groups. Independent *t*-test or nonparametric Mann–Whitney *U* test (based on normal distribution patterns) was used for comparison of continuous variables between dementia progressors and non-progressors. Chi-square test was used to compare categorical variables. All statistical analyses were performed using SPSS (version 18.0; SPSS Inc., Chicago, IL, USA). *P*-values < 0.05 were considered to indicate statistically significant differences.

## Results

### Internal validation of HCT scores

Cross-sectional assessments of test-retest reliability and inter-rater reliability showed that HCT had good intra-class correlation coefficients (test-retest Cronbach’s alpha 0.839 and inter-rater reliability Cronbach’s alpha 0.627).

### Baseline characteristics of MCI participants

A total of 38 participants diagnosed with MCI was enrolled during the study period, 32 of whom completed the follow-up cognitive evaluations to assess clinical progression. Baseline demographics, characteristics, and biomarker findings are shown in Table 1. In brief, age ranged from 55 to 80 years (mean age: 70.82 ± 5.56 years), there were more female participants (76.32%), and 33.33% were APOE4 carriers. Mean baseline K-MMSE scores was 24.79 ± 2.76 (range, 18–30). Plasma amyloid value was increased higher than the cut-off value in approximately 60% of MCI participants.

**Table 1:** Baseline characteristics and biomarker findings (MCI participants, *n* = 38)

Variables	Values
Mean age, yr	70.82 ± 5.56
Female, n (%)	29/38 (76.32%)
Education, yr	7.74 ± 3.47
Hypertension (%)	26/38 (68.42%)
Diabetes mellitus (%)	16/38 (48.48%)
Hyperlipidemia (%)	11/38 (37.93%)
APOE4 allele (%)	11/38 (33.33%)
Plasma amyloid β, ng/mL	0.80 ± 0.28
Plasma amyloid_positivity, n (%)	23/38 (60.53%)
Volumetry_frontal_lt, ml^3^	319.17 ± 1334.94
Volumetry_frontal_rt, ml^3^	73.85 ± 8.97
Volumetry_temporal_lt, ml^3^	51.75 ± 5.53
Volumetry_temporal_rt, ml^3^	48.68 ± 5.58
Volumetry_parietal_lt, ml^3^	50.07 ± 5.80
Volumetry_parietal_rt, ml^3^	51.04 ± 6.58
Volumetry_occipital_lt, ml^3^	20.58 ± 2.93
Volumetry_occipital_rt, ml^3^	21.44 ± 2.82
Volumetry_amygdala_lt, ml^3^	1.59 ± 0.27
Volumetry_amygdala_rt, ml^3^	1.74 ± 0.34
Volumetry_hippocampus_lt, ml^3^	3.99 ± 0.46
Volumetry_hippocampus_rt, ml^3^	4.21 ± 0.52
WMH, volume, ml^3^	6.11 ± 8.42
Lacune, n	0.97 ± 3.00
Microbleed, n	2.31 ± 8.52
K-MMSE total score	24.79 ± 2.76
CDR-Sum of boxes	2.03 ± 0.69
Digit span forward, percentile	72.21 ± 26.11
Boston naming, percentile	26.46 ± 26.67
RCFT copy, percentile	12.04 ± 19.02
SVLT immediate recall, percentile	18.96 ± 17.82
SVLT delayed recall, percentile	14.82 ± 16.78
SVLT recognition, percentile	28.83 ± 24.88
RCFT immediate recall, percentile	15.74 ± 14.92
RCFT delayed recall, percentile	13.08 ± 12.31
RCFT recognition, percentile	24.78 ± 19.22
COWAT phonemic, percentile	30.36 ± 26.99
Stroop Test, percentile	32.94 ± 30.70

Mean ± SD values

MCI: mild cognitive impairment, APOE4: apolipoprotein epsilon 4, WMH: white matter hyperintensities, K-MMSE: Korean version of Mini-Mental State Examination, CDR: clinical dementia rating, DST: digit span test, BNT: Boston naming test, RCFT: Rey complex figure test, SVLT: Seoul verbal learning test, COWAT: Controlled Oral Word Association Test

### Correlation analysis between HCT scores and clinical findings

**Fig. 1:**
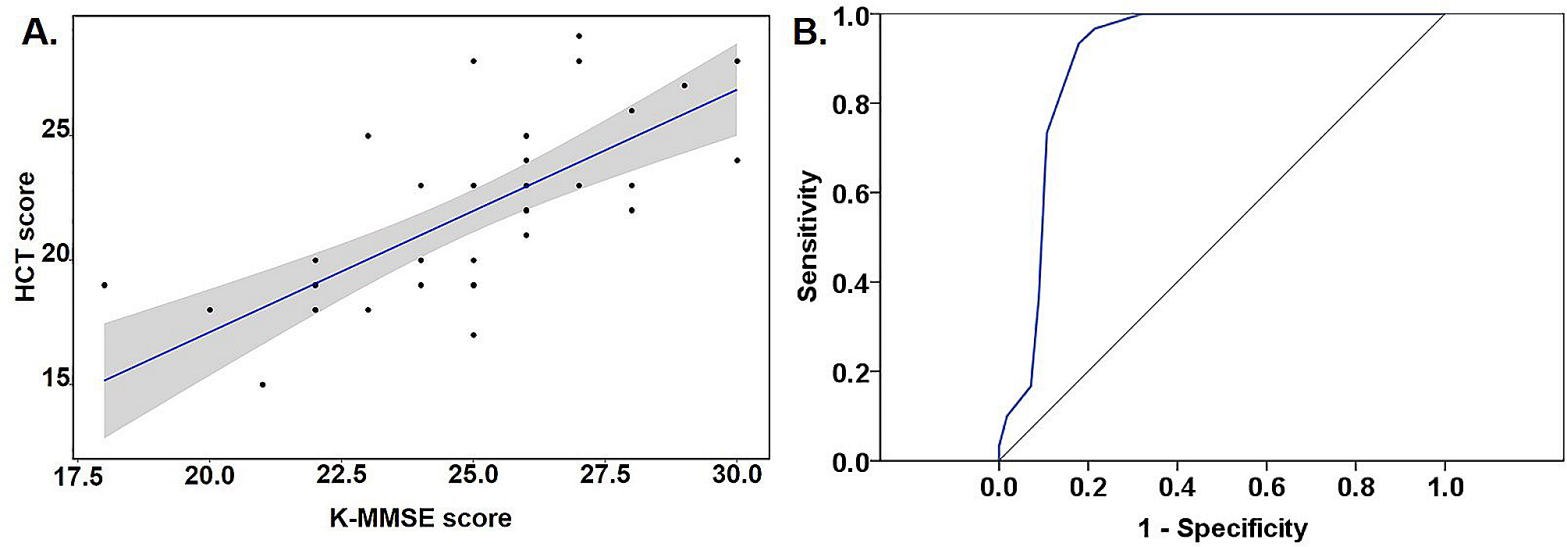
(**A**) Correlations between K-MMSE and HCT scores in MCI participants adjusted by age, gender, and education; (**B**) ROC curve showing discriminating ability of HCT between non-demented and demented subjects K-MMSE: Korean version of Mini-Mental State Examination, HCT: home-based cognitive test, MCI: mild cognitive impairment

Associations between HCT score and standard neuropsychological tests were assessed in the MCI participants. Baseline HCT score showed good correlations with baseline K-MMSE score, SVLT, and one frontal executive function test (Stroop test) after adjustment for age, sex, and education (Table 2; Fig. 1). In particular, correlation coefficient values between HCT score and K-MMSE total score were the highest (*r* = 0.780, *p* < 0.001). However, HCT scores did not show any significant associations with AD biomarkers (plasma amyloid beta values, hippocampal volumes, WMHs, lacunes, and microbleed).

**Table 2:** Correlations between HCT and in-person cognitive scores and AD biomarkers

Variables	HCT total score	
	r	*p* value
K-MMSE, total score	0.822	< 0.001*
RCFT delayed recall, percentile	0.486	0.035*
SVLT delayed recall, percentile	0.434	0.063
Stroop test, color reading, percentile	0.485	0.035*
Plasma amyloid β, ng/mL	-0.319	0.184
Volumetry_hippocampus, ml^3^	0.369	0.120
WMH, volume, ml^3^	-0.116	0.635
Lacune, *n*	-0.016	0.949
Microbleed, *n*	0.002	0.994

*Partial correlation coefficient analysis was used after adjustment for age, gender, and education

HCT: home-based cognitive test, AD: Alzheimer’s disease, K-MMSE: Korean version of Mini-Mental State Examination, RCFT: Rey complex figure test, SVLT: Seoul verbal learning test, WMH: white matter hyperintensities

### HCT scores based on clinical stage

Whether the HCT scores differed based on clinical stage (normal cognition, MCI, and mild AD dementia) was assessed. Participants with MCI did not differ in terms of demographics and comorbidities compared with normal cognition and mild AD dementia subjects (Table 3). APOE4 allele incidence, medial temporal volumes, WMH volumes, K-MMSE total scores, CDR scores, and HCT scores differed among normal cognition, MCI, and mild AD dementia subjects (*p* < 0.05, Table 3). HCT scores significantly differed based on clinical stage and was highest in participants with normal cognition and lowest in participants with AD dementia (Table 3). On ROC curves, HCT scores of 24.5 discriminated non-demented (normal cognition/MCI) and demented subjects with high sensitivity (0.933) and specificity (0.821), showing good discriminating abilities (AUC = 0.900, *p* < 0.001, Youden index = 0.75; Fig. 1).

**Table 3:** Baseline characteristics and a comparison of HCT scores according to clinical stages (normal cognition, MCI, mild AD dementia participants)

Variables	Normal cognition (*N* = 30)	MCI (*N* = 38)	AD dementia (*N* = 17)	*p*
Age, yr	69.63 ± 5.63	70.82 ± 5.56	71.65 ± 8.46	0.561
Female (%)	26 (86.67%)	29/38 (76.32%)	10 (58.82%)	0.104
Education, yr	9.73 ± 3.41	7.74 ± 3.47	8.03 ± 4.29	0.059
Hypertension	16 (53.33%)	26/38 (68.42%)	6 (35.29%)	0.089
Diabetes mellitus	11 (36.67%)	16/38 (48.48%)	5 (29.41%)	0.441
Hyperlipidemia	17 (56.67%)	11/38 (37.93%)	5 (29.41%)	0.131
APOE4 allele (%)	2 (6.67%)	11/38 (33.33%)	9 (52.94%)	0.001*
Volume_amygdala_lt, ml^3^	1.72 ± 0.20	1.59 ± 0.27	1.72 ± 0.45	0.009*
Volume_amygdala_rt, ml^3^	1.82 ± 0.28	1.74 ± 0.34	1.85 ± 0.55	0.074
Volume_hippocampus_lt, ml^3^	3.59 ± 0.35	3.99 ± 0.46	3.42 ± 0.40	0.002*
Volume_hippocampus_rt, ml^3^	3.78 ± 0.43	4.21 ± 0.52	3.62 ± 0.45	0.004*
WMH_volume, ml^3^	4.69 ± 5.56	6.11 ± 8.42	16.89 ± 16.01	0.001*
Lacune, *n*	0.43 ± 1.04	0.97 ± 3.00	2.56 ± 4.90	0.074
Microbleed, *n*	0.47 ± 1.33	2.31 ± 8.52	4.50 ± 11.72	0.232
HCT total score	26.30 ± 1.49	21.72 ± 3.69	15.24 ± 4.68	< 0.001*
K-MMSE total score	28.50 ± 1.41	24.79 ± 2.76	19.53 ± 3.62	< 0.001*
K-MMSE score_percentile	58.78 ± 15.46	22.41 ± 25.43	2.21 ± 7.14	< 0.001*
CDR-Sum of boxes	0.00 ± 0.00	2.03 ± 0.69	5.52 ± 2.15	< 0.001*

MCI: mild cognitive impairment, AD: Alzheimer’s disease, WMH: white matter hyperintensities, K-MMSE: Korean version of Mini-Mental State Examination, HCT: home-based cognitive test; CDR: clinical dementia rating

### HCT scores between progressors and non-progressors

The mean time interval between baseline and follow-up SNSB evaluations was 14.31 ± 3.49 months; range 10–25 months). A total of 32 MCI participants completed the endpoint evaluations and 8 participants (8/32, 25%) progressed to mild dementia during the study period. Baseline characteristics and clinical findings including K-MMSE scores, plasma amyloid values, small vessel disease lesions (WMHs, lacunes, microbleeds) did not differ between the progressors and non-progressors. Regional volumes including hippocampal volumes and cognitive scores such as SVLT recall and Stroop test were lower in the progressors compared with non-progressors (Supplementary Table 1). Baseline HCT scores in progressors were lower than in non-progressors (Supplementary Table 1).

## Discussion

In the present study, a telephone-based HCT tool was developed for non-demented elderly subjects and was evaluated for feasibility and use as an at-home regular cognitive monitoring tool using four assessments. First, a cross-sectional internal validation was conducted using test-retest reliability and inter-rater reliability; HCT showed good intra-class correlation coefficient values indicating consistent scores and is a feasible tool that can be repeatedly administered over the phone to elderly subjects. Second, correlation analysis was performed between HCT scores and in-person neuropsychological tests scores; HCT scores had strong positive associations with K-MMSE score and moderate positive associations with memory delayed recall scores and frontal function test. Because memory declines and frontal executive dysfunctions are early clinical signs of AD, relationships with the tests indicate the usefulness of the HCT as a monitoring tool to detect clinical progressions. Third, the discriminative ability of HCT scores based on clinical stage (normal cognition, MCI, and mild dementia) was assessed and showed that HCT scores significantly differed among normal cognition, MCI, and mild dementia subjects. Using ROC curves, HCT showed good sensitivity and specificity discriminating non-demented (normal cognition/MCI) and demented (mild dementia) status. Fourth, comparison of HCT scores between dementia progressors and non-progressors was performed. MCI participants who clinically progressed to dementia at follow-up cognitive tests showed significantly lower baseline HCT scores compared with subjects who did not progress. Because K-MMSE scores at baseline did not differ between progressors and non-progressors, HCT might be a more sensitive test to predict future clinical progression.

Due to the rapidly aging population and incidence of dementia, cognitive assessment tools that can be administered regularly and easily are necessary. However, in-person, paper and pencil type cognitive tests have the following limitations: they are difficult to perform in physically disabled patients and individuals living in rural areas; long waiting time before the tests; patient emotional burden of the in-person tests; and high cost. With the COVID-19 pandemic, many people are reluctant to visit a hospital due to fear of infection. HCTs are useful because patients can undergo cognitive tests easily and frequently without visiting the hospital, allowing monitoring of cognitive decline at home.

Remotely administered validated monitoring tools for ease of follow-up and continuity of care are increasingly necessary. Telephone-based cognitive tests are useful because most elderly subjects without dementia can easily use the telephone, can undergo cognitive tests at their convenience in a comfortable environment, do not need to read or write for the test, are cost-effective, and can overcome geographical barriers [1, 2]. Furthermore, the quick and flexible tests allow patients in the early stages of dementia (normal cognition with subjective complaints, MCI) to be regularly followed. However, previous cognitive tests via telephone have limitations in that (1) cross-sectional design [3], (2) the studies were conducted without validation using in-person neuropsychological tests [2], (3) most studies were based on White, English-speaking, and highly educated individuals [1], 4) most studies do not assess ADL independence [2], a key differentiation between MCI and dementia, 5) telephone-based test cannot evaluate visuospatial function, 6) no study examined relationships with AD-biomarkers. The HCT mitigates the limitations using detailed in-person work-ups including neuropsychological tests, neurologic examinations, and AD-biomarker findings. Our study investigated longitudinal cognitive outcomes to show that baseline HCT scores in clinical progressors were lower than in non-progressors. To alleviate the limitations, computer/tablet/or smartphone-based cognitive tests have recently been developed [3], but other limitations exist due to high-cost equipment and technological knowledge needed for the tests [3]. 

The present study had several limitations. First, the sample size was relatively small. Further studies with larger sample size might help in generalization of the results. Second, molecular imaging biomarkers such as amyloid PET or FP-CIT PET are lacking, hence, we cannot assess whether HCT is useful to predict different cognitive declines according to underlying pathologies. The last, telephone-based cognitive tests have inherent limitations such as inability to measure visuospatial functions and reading and writing abilities, and they are not suitable for subjects with hearing impairment. Therefore, in-person cognitive tests would not be discontinued, and physicians should undergo additional in-person physical, neurological, and neuropsychological examinations before confirming a diagnosis of dementia. The focus of the telephone-based HCT developed was screening and detection of early cognitive decline as a first-line monitoring tool. Future research to improve accuracy and predictability of the HCT are needed with larger sample size and sufficient biomarker findings. A smartphone-based cognitive testing may be considered to reduce examiner influence and mitigate limitations of HCT.

## Conclusions

Despite the limitations, the present study had several strengths. Participants were enrolled using a comprehensive neuropsychological test battery, and multiple biomarkers and clinical findings were used to assess reliability of the HCT. Furthermore, HCT was developed targeting Korean non-demented elderly subjects. Although further research should be performed to confirm the usefulness, the results of the present study demonstrated feasibility and reliability of the newly developed HCT. Therefore, HCT could be an alternative option to monitor cognitive decline in early stages without dementia.

### Electronic supplementary material

Below is the link to the electronic supplementary material.

Supplementary Material 1

## Data Availability

No datasets were generated or analysed during the current study.
