# Supplementary material for: Feasibility and usefulness of cognitive monitoring using a new home-based cognitive test in mild cognitive impairment: a prospective single arm study
Source: BMC Geriatr. 2024 Mar 8;24:241. doi: 10.1186/s12877-024-04850-4 (PMC10924318; doi:10.1186/s12877-024-04850-4)
Supplement: Supplementary file 1 — Supplementary Material 1 [file 12877_2024_4850_MOESM1_ESM.docx]

**Supplementary Table 1.** Baseline characteristics between dementia progressors and non-progressors (stable MCI)

| Variables | Non-progressors (N=24) | Progressors (N=8) | *p* |
| --- | --- | --- | --- |
| Age, yr | 71.83 ± 3.81 | 68.12 ± 7.14 | 0.196 |
| Female (%) | 17 (70.83%) | 7 (87.50%) | 0.642 |
| Education, yr | 8.40 ± 3.27 | 7.38 ± 3.85 | 0.469 |
| Hypertension (%) | 17 (70.83%) | 4 (50.00%) | 0.397 |
| Diabetes mellitus (%) | 13 (65.00%) | 1 (14.29%) | 0.033* |
| Hyperlipidemia (%) | 8 (40.00%) | 1 (25.00%) | 1.000 |
| APOE4 allele (%) | 7 (31.82%) | 3 (42.86%) | 0.665 |
| Plasma amyloid β, ng/ mL | 0.79 ± 0.24 | 0.85 ± 0.38 | 0.648 |
| K-MMSE total score | 25.38 ± 2.68 | 23.62 ± 3.29 | 0.141 |
| Baseline HCT total score | 23.21 ± 3.40 | 18.25 ± 2.92 | 0.001* |
| Volume_frontal_lt, ml^3^ | 71.87 ± 8.65 | 68.86 ± 10.89 | 0.491 |
| Volume_frontal_rt, ml^3^ | 73.33 ± 9.06 | 69.25 ± 10.45 | 0.362 |
| Volume_temporal_lt, ml^3^ | 50.63 ± 5.92 | 47.51 ± 4.85 | 0.255 |
| Volume_temporal_rt, ml^3^ | 48.52 ± 4.76 | 42.01 ± 5.55 | 0.010* |
| Volume_parietal_lt, ml^3^ | 50.64 ± 5.50 | 47.42 ± 6.11 | 0.235 |
| Volume_parietal_rt, ml^3^ | 51.15 ± 5.31 | 45.51 ± 5.56 | 0.035* |
| Volume_occipital_lt, ml^3^ | 19.74 ± 2.52 | 20.36 ± 3.22 | 0.623 |
| Volume_occipital_rt, ml^3^ | 20.95 ± 2.81 | 20.72 ± 2.78 | 0.859 |
| volume_amygdala_lt, ml^3^ | 1.54 ± 0.24 | 1.30 ± 0.17 | 0.037* |
| volume_amygdala_rt, ml^3^ | 1.68 ± 0.28 | 1.40 ± 0.30 | 0.047* |
| Volume_hippocampus_lt, ml^3^ | 3.30 ± 0.41 | 2.79 ± 0.31 | 0.010* |
| Volume_hippocampus_rt, ml^3^ | 3.50 ± 0.48 | 2.69 ± 0.38 | 0.001* |
| WMH volume, ml^3^ | 7.57 ± 9.99 | 6.01 ± 4.84 | 0.720 |
| Lacune, n | 1.19 ± 3.64 | 1.00 ± 1.26 | 0.227 |
| Microbleed, n | 3.38 ± 10.42 | 0.50 ± 1.22 | 0.229 |
| Digitspan Forward, percentile | 78.27 ± 21.41 | 52.20 ± 31.59 | 0.013* |
| BNT, percentile | 33.38 ± 29.21 | 11.32 ± 14.11 | 0.050 |
| RCFT copy, percentile | 9.13 ± 13.51 | 24.91 ± 32.04 | 0.214 |
| SVLT immediate recall, percentile | 21.85 ± 18.21 | 10.37 ± 11.57 | 0.106 |
| SVLT delayed recall, percentile | 18.24 ± 18.88 | 3.22 ± 3.55 | 0.001* |
| SVLT recognition, percentile | 35.04 ± 25.81 | 10.03 ± 7.86 | <0.001* |
| RCFT immediate recall, percentile | 16.13 ± 16.03 | 14.82 ± 14.98 | 0.840 |
| RCFT delayed recall, percentile | 15.49 ± 13.91 | 8.49 ± 9.07 | 0.195 |
| RCFT recognition, percentile | 27.19 ± 19.14 | 19.07 ± 22.07 | 0.325 |
| COWAT phonemic, percentile | 33.07 ± 30.78 | 22.56 ± 17.52 | 0.369 |
| Stroop test, percentile | 39.50 ± 34.14 | 14.91 ± 14.90 | 0.011* |
| Baseline CDR-Sum of boxes | 1.73 ± 0.53 | 2.81 ± 0.46 | <0.001* |

MCI: mild cognitive impairment, APOE4: apolipoprotein epsilon 4, HCT: home-based cognitive test, WMH: white matter hyperintensities, K-MMSE: Korean version of Mini-Mental State Examination, CDR: clinical dementia rating, BNT: Boston naming test, RCFT: Rey complex figure test, SVLT: Seoul verbal learning test, COWAT: Controlled Oral Word Association Test.
